# Wall Shear Stress Directional Abnormalities in BAV Aortas: Toward a New Hemodynamic Predictor of Aortopathy?

**DOI:** 10.3389/fphys.2019.00225

**Published:** 2019-03-19

**Authors:** Nimrat Grewal, Adriana C. Gittenberger-de Groot

**Affiliations:** ^1^Department of Cardiothoracic Surgery, Leiden University Medical Center, Leiden, Netherlands; ^2^Department of Anatomy and Embryology, Leiden University Medical Center, Leiden, Netherlands; ^3^Department of Cardiology, Leiden University Medical Center, Leiden, Netherlands

**Keywords:** aorta, aneurysm, bicuspid aortc valve, hemodynamic, aortopathy

We read with interest the study by Liu and coworkers in which they have addressed the role of hemodynamics in the development of aortopathy in bicuspid aortic valve (BAV) patients (Liu et al., 2018). BAV is the most common congenital cardiac malformation, with a prevalence of 1–2% in the general population (Michelena et al., 2015). Approximately half of the BAV population undergoes cardiac surgery in their lifetime due to aortopathy. Aortic dissections are eight times more likely as compared to patients with a tricuspid aortic valve (TAV) (Michelena et al., 2011). The present aortopathy treatment guidelines are based on the aortic diameter and are therefore inadequate to predict the increased risk of aortic events in the individual BAV patient (Hiratzka et al., 2016). A number of recent publications have therefore focused on identifying potential aortopathy predicting biomarkers and hemodynamic factors (Della Corte et al., 2007; Grewal et al., 2014a; Fatehi Hassanabad et al., 2019).

Janet Lui and coworkers are commendable for their effort to define the difference in wall shear stress (WSS) between BAV and TAV patients by using a new matric integrating the magnitude and angular distribution uniformity of the local WSS vector. We however do not completely agree with their suggested direct correlation between hemodynamics and aortopathy in BAV patients. As the main issue is that only a proportion of all BAV patients develop aortopathy, whereas Liu et al. performed calculations on a whole cohort.

In our earlier work we concluded that the ascending aortic wall in BAV patients is characterized by a maturation defect of the vascular smooth muscle cells (VSMCs) (Grewal et al., 2014b). As, however, the lack of differentiation of the aortic wall was seen in *all* BAV patients, it could not select those patients with an increased risk for aortic complications. In search of predictive factors for aortopathy, several studies have searched for serological and immunohistochemical factors (Schmoker et al., 2007; Tzemos et al., 2010; Black et al., 2013; Drapisz et al., 2013; Ikonomidis et al., 2013; Suzuki et al., 2013; Wang et al., 2016; Wu et al., 2016; Naito et al., 2018). We described a pathway of activated pc-Kit which distinguishes BAV patients with an increased susceptibility for future aortic wall complications. The described markers were however not related to which cusps were fused (Grewal et al., 2014a).

In search of rheologic markers, several studies revealed helical blood flow in BAV patients as compared to TAV patients, with eccentric outflow jet patterns disrupting laminar flow and flow impingement zones along the greater curvature of the ascending aorta (Girdauskas et al., 2012; Mahadevia et al., 2014a; Kimura et al., 2017).

Recent literature further focused on the hemodynamic influences for the different raphe positions in the BAV population. Normalized flow displacement is a reliable quantification of flow eccentricity as compared to systolic flow angle (Mahadevia et al., 2014b; Raghav et al., 2018). Flow displacement was greater in BAV as compared to TAV patients (Mahadevia et al., 2014b; Raghav et al., 2018). A correlation was further observed between the distal ascending aorta diameter in BAV patients with fusion of the right and non-coronary cusps (RN-BAV) but not in BAV patients with fusion of the right and left coronary cusps (RL-BAV) (Youssefi et al., 2017; Raghav et al., 2018). Flow displacement correlates with both type 1 aortopathy (involvement of the aortic root) and type 3 aortopathy (distal ascending aorta) which are more common in RN-BAV, whereas type 2 aortopathy (mid-ascending aorta) is more common in RL-BAV (Della Corte et al., 2012; Della Corte, 2014; Mahadevia et al., 2014b) as confirmed by Liu et al. (2018). In the current study the WSS data are not directly correlated to histopathologic investigation of the aortic tissue. In a recent study we investigated the role of hemodynamics in the development of aortopathy in BAV patients (Grewal et al., 2017), identifying the maximum jet stream in the ascending aorta in BAV and TAV patients. Aortic specimen were obtained during operation from the maximal jet side and the corresponding opposite non-jet side. We found a significant increase in intimal thickness on the jet side of both BAV and TAV patient groups.

An obvious increase in α smooth muscle actin positive VSMC was seen in the outer intima, whereas the inner media shows a significant decrease in expression of α smooth muscle actin of the VSMCs (Grewal et al., 2017).

We further observed a trend in transition of squamous endothelial cells in the non-jet specimen to more cuboidal endothelial cells in the jet specimen of both BAV and TAV patients. Although the 2D magnetic resonance is not appropriate to describe wall shear stress features as the flow is observed in a flat plane, we could correlate the maximum jet stream in the ascending aorta to the histopathological findings in the aortic specimen.

The main limitation of the findings of the hemodynamic studies published till date is that the WSS is observed is all BAV patients. Whereas, 20–40% of BAV patients do not develop aortic complications, implicating that the development of aortopathy in BAV cannot be explained by hemodynamic influences alone. WSS, together with the intrinsically immature aortic media which is also seen in all BAV patients, increases the vulnerability of the ascending aorta (Grewal et al., 2014b). However a second hit is necessary to cause aortopathy in selected BAV patients (Grewal et al., 2014a).

Thus, with the currently available data, a hypothesis of WSS causing aortopathy in BAV patients is hardly sustainable without ruling out the contribution of additional genetic influences.

## Author Contributions

NG: first draft of article; AG-dG: revision of article. All authors conception and design of the paper and contributed to manuscript revision, read and approved the submitted version.

### Conflict of Interest Statement

The authors declare that the research was conducted in the absence of any commercial or financial relationships that could be construed as a potential conflict of interest.
